# Establishing a precision reference benchmark for corneal epithelial aberration measurements using a new hybrid device across keratoconus stages

**DOI:** 10.1186/s40662-026-00508-x

**Published:** 2026-09-20

**Authors:** Yizhou Yang, Rui Ning, Siheng Li, Xinning Yang, Yiran Wang, Kexin Li, Giacomo Savini, Domenico Schiano-Lomoriello, Xingtao Zhou, Jinhai Huang

**Affiliations:** 1 Eye Institute and Department of Ophthalmology, NHC Key Laboratory of Myopia and Related Eye Diseases; Key Laboratory of Myopia and Related Eye Diseases, Eye & ENT Hospital, Fudan University, Chinese Academy of Medical Sciences, Shanghai, China; 2 Shanghai Research Center of Ophthalmology and Optometry, Shanghai, China; 3 Refractive Center, The Affiliated Hospital of Yunnan University, Kunming, China; 4 National Clinical Research Center for Ocular Diseases, Eye Hospital, Wenzhou Medical University, Wenzhou, 325027 China; 5 IRCCS Bietti Foundation, Rome, Italy

**Keywords:** Keratoconus, Corneal epithelial aberration, Precision, Thresholds

## Abstract

**Background:**

To establish a precision reference benchmark for corneal epithelial aberration measurements across different stages of keratoconus (KC) by evaluating the precision of a new spectral-domain optical coherence tomography–Placido disk device (MS-39; CSO).

**Methods:**

Eyes with KC were classified into forme fruste keratoconus (FFKC), mild, moderate, and severe disease stage. Three consecutive corneal epithelial aberration measurements were obtained with the MS-39 by two skilled operators. The intraobserver repeatability and interobserver reproducibility were evaluated using within-subject standard deviation (S_w_), test–retest variability (TRT), and intraclass correlation coefficient (ICC).

**Results:**

The prospective study enrolled 141 eyes from 141 KC subjects. Subgroups included 32 eyes with FFKC, 29 with mild, 45 with moderate, and 35 with severe KC. In the FFKC, mild, moderate, and total groups, S_w_ and TRT for intraobserver repeatability were ≤ 0.18 μm and ≤ 0.50 μm, respectively. ICCs were ≥ 0.884 for most parameters except trefoil and astigmatism II. The S_w_ and TRT for interobserver reproducibility were ≤ 0.15 μm and ≤ 0.41 μm, respectively. ICCs were ≥ 0.917 except for trefoil and astigmatism II. In the severe group, the ICCs for intraobserver repeatability and interobserver reproducibility were 0.366 to 0.904 and 0.493 to 0.908.

**Conclusions:**

The new MS-39 device demonstrated moderate-to-good precision in corneal epithelial aberration measurements for FFKC, mild, moderate and total KC. Despite the decrease associated with increasing KC severity, the potential of the device for early diagnosis and monitoring was still evident. The instrument’s precision should be tailored to disease severity. Stage-specific TRT thresholds for epithelial aberration measurements were proposed for KC management.

**Supplementary Information:**

The online version contains supplementary material available at https://doi.org/10.1186/s40662-026-00508-x.

## Background

Keratoconus (KC) is a degenerative disorder, characterized by a cone-shaped protrusion of the cornea [1]. Eyes with KC show a doughnut-like epithelial thickness profile, i.e., epithelial thinning over the cone surrounded by a ring of thicker epithelium [2]. As the severity of KC increases, epithelial thinning over the cone becomes more pronounced [3]. This epithelial remodeling masks irregularities of the stromal surface [4–6], making corneal epithelial aberrations a potentially relevant morphological indicator of KC [7]. Aberrations effectively describe changes in the optical properties of the eye, providing useful information for diagnosis, treatment, and follow-up. However, the understanding of corneal epithelial aberrations remains limited.

Advanced anterior segment imaging modalities have greatly enhanced our understanding and characterization of changes in KC [8]. Recently, with the growing awareness of aberrations and demand for better visual quality, an increasing number of aberrometers with novel technologies have been developed. The new hybrid MS-39 (CSO, Florence, Italy) device is able to measure epithelial aberrations, which may show earlier changes or greater sensitivity than the epithelial thickness distribution. Epithelial aberrations, as a consequence, may be used as an advanced parameter for early diagnosis, treatment and follow-up of patients with KC. Nonetheless, it is necessary to establish the precision of the MS-39 for measuring corneal epithelial aberrations. Therefore, the current study aimed to evaluate the precision of this device for measuring corneal epithelial aberrations across different grades of KC, and to establish stage-specific thresholds for clinical application.

## Methods

### Subjects

This prospective study was carried out from January 2021 to August 2023 at the Eye and ENT Hospital of Fudan University (Shanghai, China). Patients were recruited after providing written informed consent was obtained from them in accordance with the tenets of the Declaration of Helsinki.

The diagnostic criteria for KC were vision loss along with at least one biomicroscopic sign, such as focal corneal stromal thinning, Vogt’s striae, or Fleischer ring. All subjects underwent Pentacam (Oculus, Wetzlar, Germany) corneal topography measurements, which were performed by experienced operators. Corneal topography revealed one or more abnormal findings, such as maximum keratometry value (K_max_) > 47.2 diopters (D), inferior-superior difference (I-S) value > 1.4 and keratoconus percentage (KISA%) index > 100% [9–11]. Forme fruste keratoconus (FFKC) was defined as the fellow eye with KC without abnormal topography or clinical signs [12, 13]. Corneal topography demonstrated that K_max_ < 47.2 D, I-S value ≤ 1.4, KISA% index < 60% [10]. The exclusion criteria included other ocular or corneal diseases, previous ocular surgery, corneal scarring, and a recent history of wearing contact lenses (4 weeks for rigid contact lenses and 2 weeks for soft contact lenses).

Using the Topographical Keratoconus Classification (TKC) system available in the Pentacam software, all the KC subjects were categorized into “mild KC” (TKC 1 and TKC 1–2), “moderate KC” (TKC 2 and TKC 2–3) and “severe KC” (TKC 3 and TKC 3–4) while masked to all MS-39 measurement results [14]. For patients with bilateral clinically diagnosed KC who met the inclusion criteria, one eye was randomly selected using a random number table. We included only the fellow eye of patients with strictly unilateral clinically diagnosed KC, that met the pre-specified FFKC diagnostic criteria. The clinically diagnosed KC eye of the same patient was not enrolled in the study to ensure that only one eye per subject was included.

### Instrument

The MS-39 is an anterior segment device that combines spectral-domain optical coherence tomography (SD-OCT) and Placido-disk topography. It provides topography and aberration data with 31,232 measured points on the anterior surface and 25,600 measured points on the posterior surface. To acquire high-definition images, it employs an 845-nm superluminescent light-emitting diode (SLED) light source. This enables an axial resolution of 3.6 μm in tissue and a transverse resolution of 35 μm in air. For each acquisition, 25 radial scans with a 16-mm diameter are used. The interface between the epithelium and Bowman’s layer is detected by a graph search from the OCT B-scans, and the epithelial thickness measured using the MS-39 refers to the vertical distance from the tear film layer to the Bowman’s layer. A comprehensive overview of corneal epithelial aberrations is obtained in aberrometric analysis within a 6-mm pupil diameter. The net refractive index of 1.3375 was used in the calculations, and the analysis was carried out using Zernike's decomposition, which applies a set of 36 Zernike polynomials [15]. The corneal epithelial aberrations are then displayed as a Zernike polynomial map with numerical indices and graphical representations.

### Measurements

Patients were seated comfortably with their chin on the chinrest and forehead against the forehead strap in a dark room. Pupils were not dilated to avoid affecting aberrations [16]. To minimize the influence of daily corneal variations, all the measurements were conducted between 10 a.m. and 5 p.m. [17, 18]. In addition, to avoid the potential impact on epithelial thickness and epithelial aberrations measurements, the use of topical eye drops was prohibited for 2 h before the assessment [19, 20]. Before the procedure, the patients were instructed to blink sufficiently to stabilize the tear film and focus on the fixation light to prevent measurement failure caused by movement [21].

The measurements were performed by two skilled operators, each of whom performed three consecutive measurements to assess the intraobserver repeatability and interobserver reproducibility. All procedures strictly followed the manufacturer’s specifications. Only measurements with satisfactory keratoscopy acquisition quality (image coverage of 90% or greater, corneal projection center positioning of 90% or greater, and corneal projection coverage of 85% or greater), the results were included in the analysis.

The analyzed parameters included corneal epithelial total root-mean-square (RMS) aberrations, higher-order aberrations (HOAs) RMS, coma Z (3, ± 1), trefoil Z (3, ± 3), spherical aberration (SA) Z (4, 0) and astigmatism II (Ast II) Z (4, ± 2) in the central 6 mm pupil zone.

### Statistical analysis

Statistical analyses were performed using SPSS (version 21.0; IBM Corp., Armonk, NY, USA), GraphPad Prism (version 9.0; GraphPad Software, San Diego, California, USA), and MedCalc Statistical software (version 23.3; MedCalc Software Inc., Belgium). Statistical significance was defined as a two-sided *P* value < 0.05. The Kolmogorov–Smirnov test was used to assess the normality of the data, and the data were presented as mean ± standard deviation (SD). One-way ANOVA with Tukey’s honestly significant difference (HSD) post hoc test was used to compare normally distributed data among the four KC subgroups, whereas the Kruskal–Wallis H test with Bonferroni-corrected Dunn’s post-hoc test was applied for non-normally distributed parameters. Pearson’s chi-square test was used for categorical variables, with Bonferroni correction for multiple pairwise comparisons. To evaluate the repeatability and reproducibility of the instrument, the within-subject standard deviation (S_w_), the test–retest repeatability (TRT), and the intraclass correlation coefficient (ICC, two-way mixed-effects model) were used. Repeatability was independently assessed by both operators. Specifically, S_w_ was defined as the square root of the variance in a subject, and TRT was defined as 2.77 S_w_, which represented the 95% distribution range of the difference between multiple measurements. Smaller values of Sw and TRT indicated a higher repeatability and reproducibility of the measurements [22]. The ICC served as a reliability coefficient, with higher values closer to 1 indicating higher reliability. For instance, an ICC value above 0.9 indicates high reliability, whereas a value of 0.75–0.9 indicates moderate reliability, and a value below 0.4 indicates poor reliability. The Bland–Altman analysis was used to assess the agreement between paired corneal epithelial aberration measurements, with 95% limits of agreement (LoA) calculated as the mean difference ± 1.96 SD of the differences.

### Sample size calculation

For precision studies, the sample size can be calculated using S_w_, according to McAlinden et al. [23], who suggested that the sample size based on three repeated measures and a confidence in the estimate of 0.10 should exceed 96. Based on these criteria, our study met this requirement. We also performed a sample size calculation based on the expected S_w_ value of 0.15 μm obtained from our preliminary experiment, which yielded a minimum required sample size of 88 participants. Our final enrolled sample size of 141 participants met both requirements for a stable analysis.

## Results

The study enrolled 141 patients (94 males and 47 females). The demographic characteristics of the four subgroups and the total number of KC subjects are shown in Table 1. No statistically significant differences were found in age or eye laterality among the four subgroups (all *P* > 0.05), indicating well-balanced baseline characteristics. Statistically significant overall intergroup differences were detected in sex distribution, spherical equivalent (SE), cylindrical power, best-corrected visual acuity (BCVA) and K_max_ (all *P* < 0.05). Post-hoc pairwise comparisons showed significant differences in K_max_, cylindrical power, and BCVA between all pairwise subgroups (all *P* < 0.05), with K_max_ and astigmatism increasing and BCVA decreasing as KC severity increased. The sex distribution difference was mainly derived from the difference in the proportion of men between the mild and moderate KC groups (*P* = 0.013). Intraobserver repeatability was analyzed in patients stratified into four groups (32 patients in the FFKC group, 29 patients in the mild group, 45 patients in the moderate group and 35 patients in the severe group).

**Table 1:** Characteristics of four subgroups and total KC subjects

Parameter	FFKC	Mild KC	Moderate KC	Severe KC	Total KC	*P* value (4 subgroups comparison)
No. of eyes	32	29	45	35	141	–
OD/OS	18/14	15/14	20/25	18/17	71/70	0.527
Age (years)	25.06 ± 6.32	25.10 ± 6.15	26.20 ± 7.08	26.00 ± 7.21	25.67 ± 6.78	0.782
Sex (male/female)	20/12	15/14	36/9	25/10	96/45	0.018
SE (D)	− 1.25 ± 1.08	− 2.15 ± 1.82	− 3.02 ± 2.45	− 4.18 ± 2.67	− 2.78 ± 2.33	< 0.001
Cylinder (D)	− 1.05 ± 0.82	− 2.42 ± 1.58	− 3.42 ± 2.17	− 4.25 ± 2.36	−2.88 ± 2.21	< 0.001
BVCA (logMAR)	0.01 ± 0.05	0.13 ± 0.12	0.19 ± 0.15	0.41 ± 0.25	0.19 ± 0.21	< 0.001
K_max_ (D)	44.45 ± 1.87	49.68 ± 2.46	55.10 ± 4.12	67.98 ± 9.77	54.77 ± 11.24	< 0.001

*BCVA* = best-corrected visual acuity; *Cylinder* = cylindrical power; *D* = diopters; *FFKC* = forme fruste keratoconus; *K*_*max*_ = maximum keratometry; *KC* = keratoconus; *SE* = spherical equivalent

Values are presented as mean ± standard deviation

### Intraobserver repeatability

**Fig. 1:**
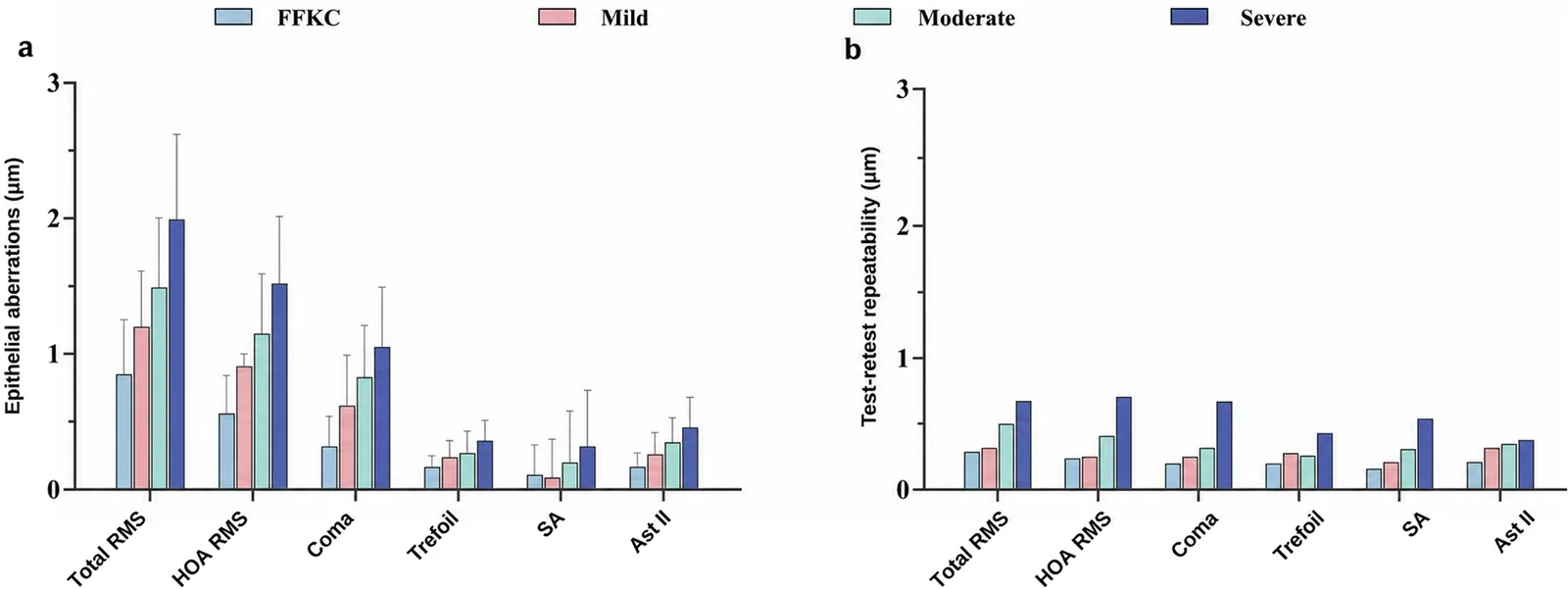
Corneal epithelial aberration measurements (**a**) and test–retest repeatability values (**b**) for the first observer across different stages of keratoconus measured using the MS-39. Ast II, astigmatism II; FFKC, forme fruste keratoconus; HOA, higher-order aberration; RMS, root mean square; SA, spherical aberration

Tables 2, 3, 4, 5, 6 show the intraobserver repeatability data corresponding to epithelial aberrations in different grades of KC and in the whole sample. Furthermore, Fig. 1 clearly demonstrates the repeatability of the first observer. The S_w_ for all types of aberrations was ≤ 0.18 μm except for the severe group, where it ranged from 0.13 to 0.28 μm, and the TRT was ≤ 0.50 μm except for the severe group, where it ranged from 0.36 to 0.77 μm. In the FFKC, mild, moderate, and total groups, the ICCs were ≥ 0.884 for most parameters except for trefoil (ICCs ranged from 0.493 to 0.797) and Ast II (ICCs ranged from 0.548 to 0.859). In the severe group, the ICCs ranged from 0.366 to 0.904.

**Table 2:** Intraobserver repeatability outcomes for corneal epithelial aberrations obtained using MS-39 in FFKC

Parameter	Observer	Mean ± SD (μm)	S_w_ (μm)	TRT (μm)	ICC (95% CI)
Total RMS	1st	0.85 ± 0.40	0.11	0.29	0.934 (0.883 to 0.966)
	2nd	0.86 ± 0.40	0.11	0.31	0.928 (0.874 to 0.962)
HOA RMS	1st	0.56 ± 0.28	0.09	0.24	0.907 (0.837 to 0.952)
	2nd	0.56 ± 0.28	0.07	0.19	0.942 (0.897 to 0.969)
Coma Z (3, ±1)	1st	0.32 ± 0.22	0.07	0.20	0.904 (0.831 to 0.950)
	2nd	0.31 ± 0.24	0.06	0.16	0.943 (0.899 to 0.970)
Trefoil Z (3, ±3)	1st	0.17 ± 0.08	0.07	0.20	0.493 (0.271 to 0.694)
	2nd	0.17 ± 0.09	0.07	0.18	0.587 (0.388 to 0.755)
SA Z (4, 0)	1st	0.11 ± 0.22	0.06	0.16	0.931 (0.878 to 0.965)
	2nd	0.13 ± 0.22	0.07	0.19	0.911 (0.846 to 0.953)
Ast II Z (4, ±2)	1st	0.17 ± 0.10	0.08	0.21	0.548 (0.334 to 0.733)
	2nd	0.16 ± 0.08	0.05	0.13	0.745 (0.593 to 0.857)

*Ast* = astigmatism; *CI* = confidence interval; *HOA* = higher-order aberration; *ICC* = intraclass correlation coefficient; *RMS* = root mean square; *SA* = spherical aberration; *SD* = standard deviation; *S*_*w*_ = within-subject standard deviation; *TRT* = test–retest repeatability (2.77 S_w_)

**Table 3:** Intraobserver repeatability outcomes for corneal epithelial aberrations obtained using MS-39 in mild KC

Parameter	Observer	Mean ± SD (μm)	S_w_ (μm)	TRT (μm)	ICC (95% CI)
Total RMS	1st	1.20 ± 0.41	0.11	0.32	0.926 (0.866 to 0.963)
	2nd	1.21 ± 0.38	0.12	0.33	0.910 (0.837 to 0.955)
HOA RMS	1st	0.91 ± 0.38	0.09	0.25	0.946 (0.901 to 0.973)
	2nd	0.91 ± 0.40	0.08	0.23	0.958 (0.921 to 0.979)
Coma Z (3, ±1)	1st	0.62 ± 0.37	0.09	0.25	0.942 (0.894 to 0.971)
	2nd	0.64 ± 0.38	0.09	0.25	0.948 (0.904 to 0.975)
Trefoil Z (3, ±3)	1st	0.24 ± 0.12	0.10	0.28	0.537 (0.313 to 0.731)
	2nd	0.24 ± 0.14	0.07	0.19	0.797 (0.653 to 0.895)
SA Z (4, 0)	1st	0.09 ± 0.28	0.08	0.21	0.930 (0.873 to 0.965)
	2nd	0.06 ± 0.26	0.07	0.19	0.932 (0.875 to 0.967)
Ast II Z (4, ±2)	1st	0.26 ± 0.16	0.12	0.32	0.609 (0.399 to 0.779)
	2nd	0.28 ± 0.15	0.08	0.21	0.788 (0.639 to 0.890)

*Ast* = astigmatism; *CI* = confidence interval; *HOA* = higher-order aberration; *ICC* = intraclass correlation coefficient; *RMS* = root mean square; *SA* = spherical aberration; *SD* = standard deviation; *S*_*w*_ = within-subject standard deviation; *TRT* = test–retest repeatability (2.77 S_w_)

**Table 4:** Intraobserver repeatability outcomes for corneal epithelial aberrations obtained using MS-39 in moderate KC

Parameter	Observer	Mean ± SD (μm)	S_w_ (μm)	TRT (μm)	ICC (95% CI)
Total RMS	1st	1.49 ± 0.51	0.18	0.50	0.884 (0.819 to 0.930)
	2nd	1.50 ± 0.47	0.15	0.43	0.900 (0.843 to 0.940)
HOA RMS	1st	1.15 ± 0.44	0.15	0.41	0.897 (0.838 to 0.938)
	2nd	1.17 ± 0.41	0.11	0.32	0.926 (0.882 to 0.956)
Coma Z (3, ±1)	1st	0.83 ± 0.38	0.11	0.32	0.916 (0.867 to 0.950)
	2nd	0.82 ± 0.38	0.11	0.31	0.917 (0.868 to 0.950)
Trefoil Z (3, ±3)	1st	0.27 ± 0.16	0.10	0.26	0.712 (0.579 to 0.817)
	2nd	0.31 ± 0.17	0.11	0.30	0.688 (0.548 to 0.801)
SA Z (4, 0)	1st	0.20 ± 0.38	0.11	0.31	0.919 (0.871 to 0.952)
	2nd	0.20 ± 0.37	0.09	0.26	0.940 (0.905 to 0.965)
Ast II Z (4, ±2)	1st	0.35 ± 0.18	0.13	0.35	0.623 (0.468 to 0.755)
	2nd	0.33 ± 0.18	0.09	0.26	0.781 (0.672 to 0.864)

*Ast* = astigmatism; *CI* = confidence interval; *HOA* = higher-order aberration; *ICC* = intraclass correlation coefficient; *RMS* = root mean square; *SA* = spherical aberration; *SD* = standard deviation; *S*_*w*_ = within-subject standard deviation; *TRT* = test–retest repeatability (2.77 S_w_)

**Table 5:** Intraobserver repeatability outcomes for corneal epithelial aberrations obtained using MS-39 in severe KC

Parameter	Observer	Mean ± SD (μm)	S_w_ (μm)	TRT (μm)	ICC (95% CI)
Total RMS	1st	1.99 ± 0.62	0.24	0.67	0.862 (0.774 to 0.922)
	2nd	2.05 ± 0.68	0.27	0.76	0.855 (0.761 to 0.919)
HOA RMS	1st	1.52 ± 0.49	0.25	0.70	0.769 (0.638 to 0.866)
	2nd	1.62 ± 0.63	0.28	0.77	0.829 (0.722 to 0.904)
Coma Z (3, ±1)	1st	1.05 ± 0.44	0.24	0.67	0.754 (0.616 to 0.856)
	2nd	1.06 ± 0.47	0.20	0.54	0.843 (0.743 to 0.912)
Trefoil Z (3, ±3)	1st	0.36 ± 0.15	0.15	0.43	0.366 (0.157 to 0.576)
	2nd	0.41 ± 0.27	0.20	0.55	0.605 (0.419 to 0.760)
SA Z (4, 0)	1st	0.32 ± 0.41	0.19	0.54	0.807 (0.692 to 0.890)
	2nd	0.30 ± 0.42	0.14	0.38	0.904 (0.838 to 0.947)
Ast II Z (4, ±2)	1st	0.46 ± 0.22	0.14	0.38	0.703 (0.547 to 0.824)
	2nd	0.47 ± 0.33	0.13	0.36	0.859 (0.767 to 0.921)

*Ast* = astigmatism; *CI* = confidence interval; *HOA* = higher-order aberration; *ICC* = intraclass correlation coefficient; *RMS* = root mean square; *SA* = spherical aberration; *SD* = standard deviation; *S*_*w*_ = within-subject standard deviation; *TRT* = test–retest repeatability (2.77 S_w_)

**Table 6:** Intraobserver repeatability outcomes for corneal epithelial aberrations obtained using MS-39 in total KC

Parameter	Observer	Mean ± SD (μm)	S_w_ (μm)	TRT (μm)	ICC (95% CI)
Total RMS	1st	1.42 ± 0.65	0.18	0.49	0.929 (0.907 to 0.947)
	2nd	1.44 ± 0.66	0.18	0.50	0.930 (0.908 to 0.947)
HOA RMS	1st	1.07 ± 0.53	0.16	0.45	0.911 (0.884 to 0.933)
	2nd	1.09 ± 0.58	0.16	0.45	0.928 (0.906 to 0.946)
Coma Z (3, ±1)	1st	0.74 ± 0.45	0.15	0.41	0.899 (0.868 to 0.924)
	2nd	0.73 ± 0.46	0.13	0.35	0.928 (0.905 to 0.946)
Trefoil Z (3, ±3)	1st	0.27 ± 0.15	0.11	0.31	0.599 (0.510 to 0.681)
	2nd	0.29 ± 0.20	0.13	0.35	0.693 (0.617 to 0.760)
SA Z (4, 0)	1st	0.19 ± 0.35	0.13	0.35	0.883 (0.848 to 0.911)
	2nd	0.18 ± 0.35	0.10	0.27	0.925 (0.902 to 0.944)
Ast II Z (4, ±2)	1st	0.32 ± 0.20	0.12	0.33	0.719 (0.648 to 0.782)
	2nd	0.32 ± 0.24	0.09	0.26	0.859 (0.818 to 0.893)

*Ast* = astigmatism; *CI* = confidence interval; *HOA* = higher-order aberration; *ICC* = intraclass correlation coefficient; *RMS* = root mean square; *SA* = spherical aberration; *SD* = standard deviation; *S*_*w*_ = within-subject standard deviation; *TRT* = test–retest repeatability (2.77 S_w_)

### Interobserver reproducibility

**Fig. 2:**
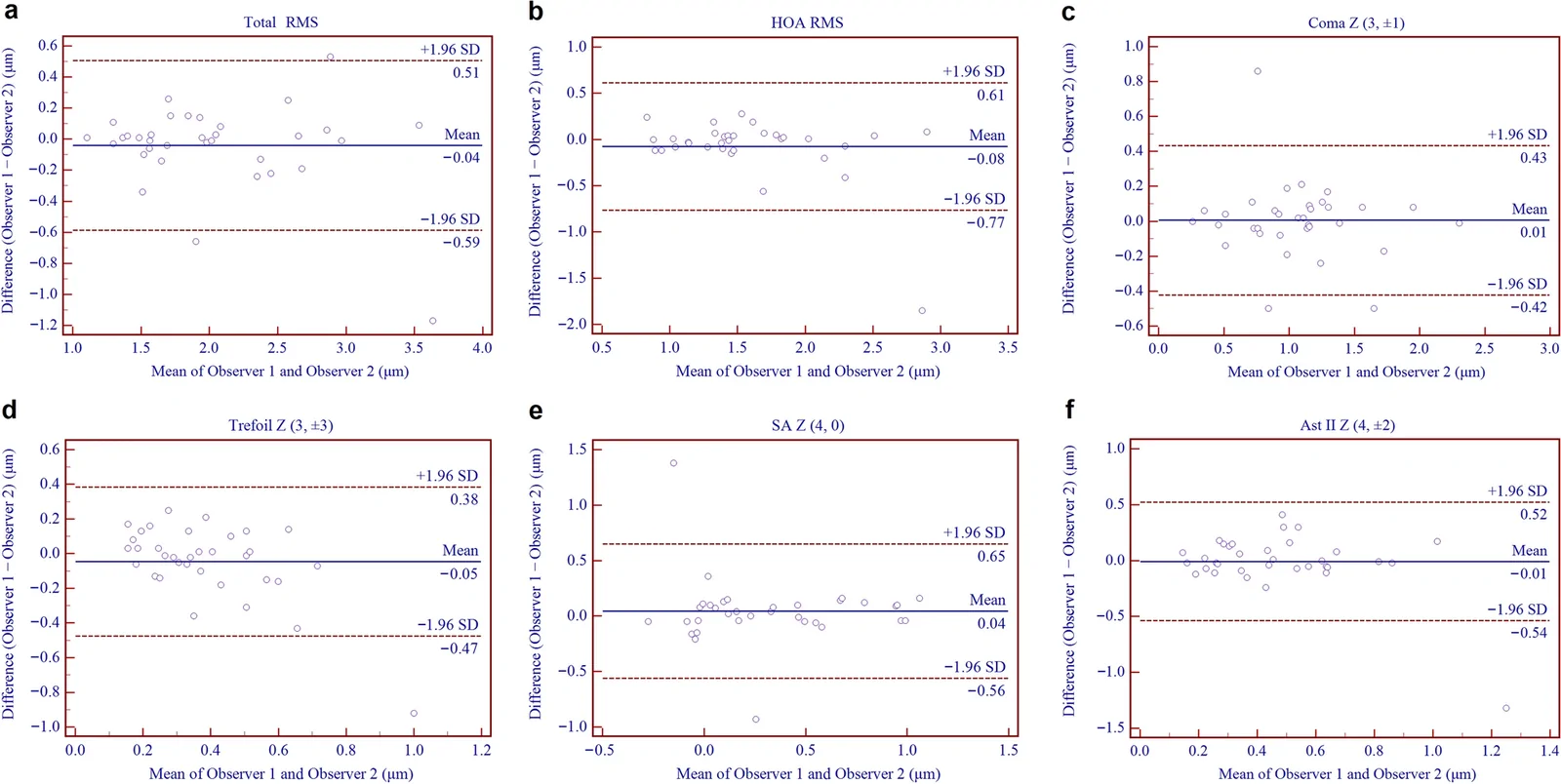
Bland–Altman plots showing agreement between observers 1 and 2 for measurements of total RMS (**a**), HOA RMS (**b**), coma (**c**), trefoil (**d**), SA (**e**) and Ast II (**f**) in the severe keratoconus group. Ast II, astigmatism II; HOA, higher-order aberration; RMS, root mean square; SA, spherical aberration; SD, standard deviation

Supplementary tables 1–5 display the interobserver reproducibility of the MS-39 measurements across different grades of KC and in the overall sample. The S_w_ of all the parameters was ≤ 0.15 μm except for the severe group, where it ranged from 0.15 to 0.25 μm, and the TRT was ≤ 0.41 μm except for the severe group, where it ranged from 0.42 to 0.69 μm. In the FFKC, mild, moderate, and total groups, the ICCs were ≥ 0.917 for most parameters except for trefoil (ICCs ranged from 0.705 to 0.838) and Ast II (ICCs ranged from 0.772 to 0.889). In the severe group, the ICCs ranged from 0.493 to 0.908. Bland–Altman plots were generated to illustrate the variability of epithelial aberration parameters in the severe KC group, as shown in Fig. 2.

## Discussion

Early detection of KC remains challenging. Bowman’s layer, located just above the stroma, experiences significant thinning in keratoconic eyes [24], which is also associated with disease-related alterations [25]. Previous studies have analyzed the three-dimensional form of the corneal epithelium and stroma in KC [7, 26, 27] and reported that the curvature of Bowman’s layer is steeper than that of the curvature of the corneal anterior surface. Corneal epithelial aberrations arise from compensatory epithelial remodeling in response to stromal protrusion in KC and may indirectly reflect secondary morphological and curvature changes in Bowman’s layer beneath the epithelial layer. Therefore, corneal epithelial aberrations may serve as earlier and more sensitive markers than anterior corneal aberrations.

Consequently, the high-resolution OCT imaging of Bowman’s layer can serve as a biomarker for early detection of ectasia, and monitoring corneal epithelial aberrations may be an appealing alternative for the follow-up of patients with KC. To the best of our knowledge, few studies have analyzed the corneal epithelial aberrations parameters obtained using MS-39. Repeatable and reproducible measurements are important in clinical practice. The aim of the current study was to comprehensively evaluate the precision of MS-39 epithelial aberration measurements across the different stages of KC and to establish a stage-specific precision benchmark for clinical application.

In our study, corneal epithelial aberrations generally showed a gradual increase as KC severity increased. The S_w_ values showed a similar trend. The ICCs gradually decreased with increasing KC severity, except for the trefoil and Ast II groups. The repeatability of trefoil and Ast II did not change apparently in different stages of KC. In addition, the repeatability of trefoil and Ast II was not as stable as that of the other parameters; the ICCs ranged from 0.366 to 0.797 and from 0.548 to 0.859, respectively, in different grades of KC. With regard to the measurements reproducibility, the results were more acceptable with all ICCs ≥ 0.772, except for trefoil in the FFKC, severe and total groups (ICC = 0.705, 0.493 and 0.723), SA in the severe group (ICC = 0.73) and Ast II in the severe group (ICC = 0.558). As described above, the epithelial thickness measured by MS-39 represents the vertical distance between the tear film and Bowman’s layer. SD-OCT provides depth-resolved tissue structural information encoded by the magnitude and delay of backscattered light through spectral analysis. Edge detection within hierarchical structure of the cornea is essential. However, as KC severity progresses, the epithelial thinning may pose challenges to edge detection, leading to reduced measurement accuracy.

Notably, the statistical characteristics of the ICC must be considered when interpreting these results. The ICC value reflects the proportion of total variance attributed to true intersubject biological differences, and is therefore influenced by random measurement error and the degree of variability within the study sample. For parameters with inherently low numerical dispersion, such as trefoil and Ast II in early KC, even a minimal absolute measurement error may result in a moderate or even low ICC value, which does not necessarily indicate clinically insufficient measurement precision. Therefore, in addition to ICC analysis, we prioritized TRT as the primary endpoint for assessing clinical applicability in this precision study. The TRT is mathematically defined as the minimal detectable change at the 95% confidence level in precision research, which represents the smallest parameter change that exceeds random measurement error.

Currently few studies have focused on corneal epithelial aberrations. Matalia et al. [15] conducted a study on aberrations at the air–epithelium and epithelium–Bowman’s layer interfaces. They used Triton OCT (Topcon, Tokyo, Japan) for epithelial HOA measurements and reported excellent repeatability, with ICCs greater than 0.90. The S_w_ values of HOA RMS were 0.07 μm and 0.10 μm at air–epithelium and epithelium–Bowman’s layer interfaces, corresponding to TRT values of 0.19 μm and 0.28 μm. Similarly, the S_w_ values of both horizontal coma and vertical coma were 0.04 μm, with a corresponding TRT of 0.11 μm. For SA, the S_w_ values were 0.02 μm and 0.03 μm, with corresponding TRT values of 0.06 μm and 0.08 μm. However, they did not analyze the thresholds. Furthermore, there are fundamental differences between this prior work and our study, which limit direct comparisons. The aberration metrics they obtained were derived from the elevation data of a single rigid optical interface, whereas our study quantified aberrations across the entire corneal epithelial layer, which inherently incorporates the physiological spatial variation of epithelial thickness and disease-related compensatory remodeling. Moreover, they did not include analyses of trefoil and Ast II or classify KC patients; their cohort was predominantly composed of mild to moderate KC. Many studies have suggested that trefoil and coma primarily reflect corneal asymmetry, which has diagnostic significance for KC, a condition characterized by asymmetric corneal expansion [28]. Therefore, we believe that understanding the performance of the MS-39 in measuring trefoil has meaningful implications for KC. However, the repeatability of the trefoil in our study was relatively poor and unstable. This may be attributable to significant stromal protrusion and epithelium thinning. These changes lead to marked corneal irregularity and tear film instability, which significantly increase measurement error and difficulty. Overall, for the FFKC, mild, moderate, and total KC groups, the repeatability was mostly moderate to good, demonstrating the potential of the instrument for the early diagnosis and follow-up of KC.

Given that KC is a progressive disease with distinct structural and optical changes across stages, stage-specific TRT thresholds are warranted to guide clinical interpretation. For FFKC, mild, moderate, and total KC groups, TRT values for all epithelial aberration parameters met a uniform clinical threshold of ≤ 0.50 µm for both intraobserver repeatability and interobserver reproducibility. This indicates that changes exceeding 0.50 µm in these subgroups can be reliably interpreted as biological changes of corneal epithelial remodeling, rather than random measurement error. For the precision analysis of total RMS, HOA RMS, coma, trefoil, SA, and Ast II within the 6 mm pupil zone, we propose stage-specific TRT thresholds as follows: 0.31, 0.24, 0.20, 0.20, 0.19, and 0.21 µm for FFKC; 0.33, 0.25, 0.25, 0.28, 0.21, and 0.32 µm for mild KC; and 0.50, 0.41, 0.32, 0.30, 0.31, and 0.35 µm for moderate KC. These thresholds represent the minimum detectable changes for each parameter across different KC grades, and careful clinical interpretation is required once the measured changes exceed these thresholds. Collectively, these findings confirm the clinical utility of the MS-39 device for the early detection of KC and monitoring its longitudinal progression. In the severe KC group, the TRT values for some parameters were markedly elevated, ranging from 0.36 µm to 0.77 µm for intraobserver repeatability and from 0.42 µm to 0.69 µm for interobserver reproducibility. The Bland–Altman plots further confirmed that there was no clinically significant systematic bias between paired measurements (mean difference < 0.1 μm for all parameters, with the 95% confidence intervals for the mean difference all crossing zero), demonstrating that the reduced reliability was driven by increased random measurement error, rather than operator-related systematic bias. The rare outliers observed in the Bland–Altman plots may be attributable to extreme corneal irregularities in advanced cases. These findings provide direct guidance for clinical practice, indicating that the MS-39 device is not recommended as the sole basis for clinical decision-making in patients with severe KC. Extreme caution should be exercised when interpreting these measurements in patients with advanced KC, with careful distinction between true disease-related changes and random measurement variability.

With wide radial scans and a measurement depth of up to 7 mm, the MS-39 can directly acquire corneal epithelial aberrations without removing the epithelium. As it is widely known, whether the epithelium is mechanically removed or chemically dissolved, the morphology of Bowman’s layer is affected [29, 30]. This may explain why previous corneal topographers, such as Pentacam, were prone to measurement errors. Moreover, Georgeon et al. [31] found that the reproducibility of peripheral corneal epithelial thickness measurements was better than that of other SD-OCT devices. Therefore, the use of MS-39 may facilitate the detection of peripheral doughnut-like patterns and the identification of other ectasia diseases, such as pellucid marginal degeneration.

A limitation of our study is that no other principle-based devices were included, and the presence of device-specific differences in epithelial and Bowman’s layer metrics across OCT platforms may affect the clinical application [32]. In future studies, we intend to address this limitation by including patients with more severe KC, as well as other types of corneal ectasia diseases, thereby enhancing the comprehensiveness of the research findings. Another limitation of this study was that objective tear film metrics were not quantitatively assessed to confirm tear film stability. Although all measurements were acquired under strict image quality criteria to exclude unstable conditions, the lack of an objective assessment represents a minor methodological limitation.

## Conclusions

In conclusion, this is the first study to systematically investigate the measurement precision of corneal epithelial aberrations in patients with KC stratified by disease severity using the MS-39 and to establish stage-specific thresholds for clinical interpretation. Moderate-to-good repeatability and reproducibility were observed in the FFKC, mild, moderate, and total KC groups. The stage-specific TRT thresholds proposed in this study provide a clinical reference for interpreting epithelial aberration measurements, supporting the potential of MS-39 for early diagnosis and monitoring of KC progression. However, clinical interpretation of these measurements in patients with advanced KC requires extreme caution.

## Supplementary Information

Supplementary material 1.

## Data Availability

All data generated or analysed during this study are included in this published article.

## Abbreviations

KC: Keratoconus
FFKC: Forme fruste keratoconus
K_max_: Maximum keratometry
I-S value: Inferior-superior difference value
KISA% index: Keratoconus percentage index
TKC: Topographical Keratoconus Classification
SE: Spherical equivalent
D: Diopters
BCVA: Best-corrected visual acuity
SD-OCT: Spectral-domain optical coherence tomography
SLED: Superluminescent light-emitting diode
RMS: Root mean square
HOA: Higher-order aberration
SA: Spherical aberration
Ast II: Astigmatism II
SD: Standard deviation
S_w_: Within-subject standard deviation
TRT: Test–retest repeatability
ICC: Intraclass correlation coefficient
LOA: Limits of agreement
