# Supplementary material for: Establishing a precision reference benchmark for corneal epithelial aberration measurements using a new hybrid device across keratoconus stages
Source: Eye Vis (Lond). 2026 Sep 20;13:38. doi: 10.1186/s40662-026-00508-x (PMC13589856; doi:10.1186/s40662-026-00508-x)
Supplement: Supplementary file 1 [file 40662_2026_508_MOESM1_ESM.docx]

| Supplemental Table 1. Interobserver reproducibility outcomes for corneal epithelial aberrations obtained using MS-39 in forme fruste keratoconus | | | | |
| --- | --- | --- | --- | --- |
| Parameter | Mean ± SD (μm) | S_w_ (μm) | TRT (μm) | ICC (95% CI) |
| Total RMS | 0.85 ± 0.39 | 0.08 | 0.21 | 0.965 (0.927 to 0.983) |
| HOA RMS | 0.55 ± 0.27 | 0.05 | 0.15 | 0.963 (0.922 to 0.982) |
| Coma Z (3, ±1) | 0.32 ± 0.23 | 0.04 | 0.11 | 0.970 (0.937 to 0.986) |
| Trefoil Z (3, ±3) | 0.17 ± 0.08 | 0.05 | 0.13 | 0.705 (0.461 to 0.850) |
| SA Z (4, 0) | 0.12 ± 0.21 | 0.06 | 0.17 | 0.923 (0.843 to 0.963) |
| Ast II Z (4, ±2) | 0.17 ± 0.08 | 0.04 | 0.11 | 0.794 (0.607 to 0.898) |
| Ast = astigmatism; CI = confidence interval; HOA = higher-order aberration; ICC = intraclass correlation coefficient; RMS = root mean square; SA = spherical aberration; SD = standard deviation; S_w_ = within-subject standard deviation; TRT = test-retest repeatability (2.77 S_w_) | | | | |

| Supplemental Table 2. Interobserver reproducibility outcomes for corneal epithelial aberrations obtained using MS-39 in mild keratoconus | | | | | |
| --- | --- | --- | --- | --- | --- |
| Parameter | Mean ± SD (μm) | S_w_ (μm) | TRT (μm) | ICC (95% CI) | |
| Total RMS | 1.22 ± 0.4 | 0.10 | 0.28 | 0.935 (0.862 to 0.97) | |
| HOA RMS | 0.92 ± 0.39 | 0.07 | 0.19 | 0.970 (0.935 to 0.986) | |
| Coma Z (3, ±1) | 0.64 ± 0.37 | 0.06 | 0.16 | 0.976 (0.948 to 0.989) | |
| Trefoil Z (3, ±3) | 0.24 ± 0.13 | 0.05 | 0.15 | 0.838 (0.676 to 0.923) | |
| SA Z (4, 0) | 0.07 ± 0.27 | 0.06 | 0.17 | 0.945 (0.883 to 0.975) | |
| Ast II Z (4, ±2) | 0.28 ± 0.16 | 0.05 | 0.15 | 0.881 (0.756 to 0.944) | |
| Ast = astigmatism; CI = confidence interval; HOA = higher-order aberration; ICC = intraclass correlation coefficient; RMS = root mean square; SA = spherical aberration; SD = standard deviation; S_w_ = within-subject standard deviation; TRT = test-retest repeatability (2.77 S_w_) | | | | |  |

| Supplemental Table 3. Interobserver reproducibility outcomes for corneal epithelial aberrations obtained using MS-39 in moderate keratoconus | | | | | |
| --- | --- | --- | --- | --- | --- |
| Parameter | Mean ± SD (μm) | S_w_ (μm) | TRT (μm) | ICC (95% CI) | |
| Total RMS | 1.49 ± 0.48 | 0.12 | 0.33 | 0.940 (0.894 to 0.967) | |
| HOA RMS | 1.16 ± 0.42 | 0.12 | 0.34 | 0.917 (0.853 to 0.953) | |
| Coma Z (3, ±1) | 0.83 ± 0.38 | 0.09 | 0.25 | 0.942 (0.898 to 0.968) | |
| Trefoil Z (3, ±3) | 0.29 ± 0.16 | 0.07 | 0.20 | 0.824 (0.701 to 0.899) | |
| SA Z (4, 0) | 0.20 ± 0.37 | 0.07 | 0.20 | 0.962 (0.932 to 0.979) | |
| Ast II Z (4, ±2) | 0.34 ± 0.18 | 0.06 | 0.17 | 0.889 (0.807 to 0.938) | |
| Ast = astigmatism; CI = confidence interval; HOA = higher-order aberration; ICC = intraclass correlation coefficient; RMS = root mean square; SA = spherical aberration; SD = standard deviation; S_w_ = within-subject standard deviation; TRT = test-retest repeatability (2.77 S_w_) | | | | |  |

| Supplemental Table 4. Interobserver reproducibility outcomes for corneal epithelial aberrations obtained using MS-39 in severe keratoconus | | | | |
| --- | --- | --- | --- | --- |
| Parameter | Mean ± SD (μm) | S_w_ (μm) | TRT (μm) | ICC (95% CI) |
| Total RMS | 2.01 ± 0.64 | 0.20 | 0.54 | 0.908 (0.826 to 0.953) |
| HOA RMS | 1.56 ± 0.54 | 0.25 | 0.69 | 0.804 (0.645 to 0.896) |
| Coma Z (3, ±1) | 1.05 ± 0.44 | 0.15 | 0.42 | 0.890 (0.792 to 0.943) |
| Trefoil Z (3, ±3) | 0.38 ± 0.19 | 0.15 | 0.43 | 0.493 (0.195 to 0.707) |
| SA Z (4, 0) | 0.30 ± 0.39 | 0.21 | 0.59 | 0.730 (0.528 to 0.854) |
| Ast II Z (4, ±2) | 0.47 ± 0.25 | 0.19 | 0.51 | 0.558 (0.281 to 0.750) |
| Ast = astigmatism; CI = confidence interval; HOA = higher-order aberration; ICC = intraclass correlation coefficient; RMS = root mean square; SA = spherical aberration; SD = standard deviation; S_w_ = within-subject standard deviation; TRT = test-retest repeatability (2.77 S_w_) | | | | |

| Supplemental Table 5. Interobserver reproducibility outcomes for corneal epithelial aberrations obtained using MS-39 in total keratoconus | | | | |
| --- | --- | --- | --- | --- |
| Parameter | Mean ± SD (μm) | S_w_ (μm) | TRT (μm) | ICC (95% CI) |
| Total RMS | 1.42 ± 0.64 | 0.13 | 0.37 | 0.958 (0.942 to 0.970) |
| HOA RMS | 1.07 ± 0.55 | 0.15 | 0.41 | 0.929 (0.902 to 0.949) |
| Coma Z (3, ±1) | 0.73 ± 0.45 | 0.10 | 0.27 | 0.954 (0.936 to 0.967) |
| Trefoil Z (3, ±3) | 0.28 ± 0.16 | 0.09 | 0.26 | 0.723 (0.632 to 0.794) |
| SA Z (4, 0) | 0.18 ± 0.34 | 0.12 | 0.34 | 0.874 (0.828 to 0.909) |
| Ast II Z (4, ±2) | 0.32 ± 0.21 | 0.10 | 0.29 | 0.772 (0.695 to 0.832) |
| Ast = astigmatism; CI = confidence interval; HOA = higher-order aberration; ICC = intraclass correlation coefficient; RMS = root mean square; SA = spherical aberration; SD = standard deviation; S_w_ = within-subject standard deviation; TRT = test-retest repeatability (2.77 S_w_) | | | | |
